# Single-Cell Transcriptomes Reveal a Complex Cellular Landscape in the Middle Ear and Differential Capacities for Acute Response to Infection

**DOI:** 10.3389/fgene.2020.00358

**Published:** 2020-04-15

**Authors:** Allen F. Ryan, Chanond A. Nasamran, Kwang Pak, Clara Draf, Kathleen M. Fisch, Nicholas Webster, Arwa Kurabi

**Affiliations:** ^1^Departments of Surgery/Otolaryngology, UC San Diego School of Medicine, VA Medical Center, La Jolla, CA, United States; ^2^Medicine/Center for Computational Biology & Bioinformatics, UC San Diego School of Medicine, VA Medical Center, La Jolla, CA, United States; ^3^Medicine/Endocrinology, UC San Diego School of Medicine, VA Medical Center, La Jolla, CA, United States

**Keywords:** single-cell, middle ear, otitis media, cluster-profiling, homeostasis

## Abstract

Single-cell transcriptomics was used to profile cells of the normal murine middle ear. Clustering analysis of 6770 transcriptomes identified 17 cell clusters corresponding to distinct cell types: five epithelial, three stromal, three lymphocyte, two monocyte, two endothelial, one pericyte and one melanocyte cluster. Within some clusters, cell subtypes were identified. While many corresponded to those cell types known from prior studies, several novel types or subtypes were noted. The results indicate unexpected cellular diversity within the resting middle ear mucosa. The resolution of uncomplicated, acute, otitis media is too rapid for cognate immunity to play a major role. Thus innate immunity is likely responsible for normal recovery from middle ear infection. The need for rapid response to pathogens suggests that innate immune genes may be constitutively expressed by middle ear cells. We therefore assessed expression of innate immune genes across all cell types, to evaluate potential for rapid responses to middle ear infection. Resident monocytes/macrophages expressed the most such genes, including pathogen receptors, cytokines, chemokines and chemokine receptors. Other cell types displayed distinct innate immune gene profiles. Epithelial cells preferentially expressed pathogen receptors, bactericidal peptides and mucins. Stromal and endothelial cells expressed pathogen receptors. Pericytes expressed pro-inflammatory cytokines. Lymphocytes expressed chemokine receptors and antimicrobials. The results suggest that tissue monocytes, including macrophages, are the master regulators of the immediate middle ear response to infection, but that virtually all cell types act in concert to mount a defense against pathogens.

## Introduction

The middle ear (ME) is a bone-encased, air-filled cavity that links the external ear to the inner ear. Bounded externally by the tympanic membrane, it houses the three ossicles that transmit acoustic vibrations from the eardrum to the cochlea. It is connected to the nasopharynx by the Eustachian tube, which provides intermittent ventilation and pressure release, as well as clearance via ciliary activity.

The ME is a frequent site of infection, especially in children. More than 90% of children experience otitis media (OM), which is chronic-recurrent in 15−20% (Rosenfeld and Bluestone, 2003). It is the most common cause of physician visits and surgery at ages less than 5 years (Cullen et al., 2009). OM leads to hearing loss during a critical period of language acquisition and learning, and has been associated with deficits in both language and learning (Friel-Patti et al., 1982; Wendler-Shaw et al., 1993). OM can be well-controlled by therapy, but the annual cost in the United States is estimated at >$5 billion (Ahmed et al., 2014). In contrast, OM is a very serious disease in developing countries due to limited access to medical care. Under-treated suppurative OM is estimated by the World Health Organization to be responsible for 28,000 annual deaths due to intracranial infection and to cause half of the world’s burden of severe hearing loss, approximately 240 million cases (Arguedas et al., 2010). The resolution of uncomplicated, acute OM occurs in less than one week, even without antibiotic treatment. This is too rapid for the *de novo* elaboration of cognate immunity, suggesting that OM is normally resolved by innate immunity. Indeed, deficiencies in innate immune genes have been linked to OM susceptibility in both mice and humans (Leichtle et al., 2011; Rye et al., 2011).

The ME is an atypical mucosal site, characterized by a largely rudimentary cellular structure, yet with the capability to rapidly transform into a respiratory-type epithelium. The majority of the resting ME cavity is lined by a monolayer of simple squamous epithelial cell overlying a sparse stroma and vasculature. However, upon infection and inflammation, hyperplasia can produce a 20-fold increase in thickness, into a pseudostratified, columnar epithelium populated with ciliated, goblet and secretory cells, within a few days. Upon the resolution of infection, the mucosa returns to its baseline structure (Lim, 1979).

The cells that make up the normal ME mucosa have been studied primarily morphologically. Early studies were limited to the observation of a simple squamous lining epithelium with ciliated cells in some areas, especially near the orifice of the Eustachian tube (e.g., Kolmer and Mellendorff, 1927; Wolff, 1943). Later investigations noted morphological details that confirmed a simple squamous epithelial structure with minimal stroma throughout much of the ME, but with some areas of cuboidal, columnar and pseudostratified epithelium, primarily near the Eustachian tube but also in recesses and corners of the ME (e.g., Hentzer, 1970). Blood vessels, lymphatics, and small numbers of immunocytes including macrophages, lymphocytes and plasma cells were also noted in the stroma, as were melanocytes (Lin and Zak, 1982). In addition, histochemistry was used to demonstrate the presence of mucopolysaccharides within both ciliated and non-ciliated epithelial cells (e.g., Sade, 1966).

The introduction of electron microscopy to ME studies added significant details regarding ME cell types. Based on their ultrastructure, Lim and Hussl (1969) classified ME epithelial cells as non-ciliated without secretory granules, mucus-secreting goblet cells with abundant secretory granules, intermediary secretory cells with fewer secretory granules, ciliated cells, and subsurface basal cells. Hentzer (1976) added a sixth class that he termed intermediate cells, which he proposed could differentiate into any of the other surface cell types, with intermediary secretory cells a transitional stage into goblet cells. Ultrastructural studies also noted pericytes in association with the endothelial cells of vessels.

Additional information has been added by immunohistochemistry. Takahashi et al. (1989) identified macrophages, T-cells and B-cells in the subepithelial stroma of the normal ME. However, these cells were sparse, with on average less than one B cell in a histological section through the entire ME, and 1−5 macrophages and T-cells per section. They thus comprised a very small fraction of the cells present in the normal ME, as observed morphologically by Lim (1979). Immunohistochemistry was also used to document the presence of melanocytes (Lin and Zak, 1982), pericytes (Zhang et al., 2015), natural killer cells (Jecker et al., 1996), and mast cells (Stenfors et al., 1985).

More recently, a lineage study found that the ME mucosal epithelium in mice has two distinct embryonic origins. Epithelial cells in approximately half of the ME cavity closest to the Eustachian tube orifice originate from the endoderm of the branchial arches, while the remainder originates from the neural crest. In the normal ME, ciliated and goblet cells were observed only in cells of endodermal origin, although many non-ciliated, non-secretory cells were also present (Thompson and Tucker, 2013). More recently, ciliated cells have been documented in the region of neural crest origin (Luo et al., 2017). However, the dual origin of cells adds yet another layer of complexity to ME cell types.

Recent studies have also evaluated the transcriptome of the normal ME mucosa (MacArthur et al., 2013; Hernandez et al., 2015). These studies, which utilized bulk RNA, identified genes expressed in the normal ME. However, they could not address the genes expressed individually by the different ME cell types. Cell-specific patterns of genes expression would help to define the functions of various cell types and their ability to respond to ME infection.

The purpose of the present study was to document the expression of genes in individual ME cells. We sought to identify the cell types present in the ME cavity at a single-cell resolution, to illuminate their functional characteristics, and to evaluate the cellular distribution of genes known to be important for the pathogenesis and resolution of OM.

## Materials and Methods

### Animals

Young adult (60−90 day old) wildtype C57Bl/6J mice (Charles River, Wilmington, MA, United States) were used. All procedures were performed to National Institutes of Health guidelines and approved by the Institutional Animal Care and Use Committee of the VA San Diego Medical Center.

### Preparation of Cell Suspensions

Groups of six mice were deeply anesthetized (ketamine 50 mg/kg, xylazine 1 mg/kg, acepromazine 5 mg/kg in 50 μl, i.p.) and sacrificed by decapitation, avoiding significant pressure on the neck to prevent the rupture of blood vessels in the ME. Six animals were required to generate a sufficient number of cells for a 10X Genomics run, because a ME tissue sample from a single animal are small. The ME bullae were isolated and opened along the suture that divides the lateral from the medial ME. ME mucosal tissue was gently harvested from the bullar bone. Care was taken not to include any tissue from the ME muscles. The pooled tissue was incubated with 0.5 mg/ml thermolysin (Sigma-Aldrich, #T7902) in Leibovitz’s buffer for 25–30 min in a 37°C/5% CO_2_ humidified tissue culture incubator to dissociate the extracellular matrix. The thermolysin was then aspirated, the tissue rinsed, and the sample incubated in FACSMax cell dissociation solution (Genlantis, #T200100). The cell mixture was triturated with a pipette and further dissociated into single cells mechanically by passing through a 23 G blunt-ended needle. Dissociated cells were passed through a 40 μm cell strainer (BD Biosciences) to eliminate clumps before sorting and collected into a FACS tube on ice containing PBS buffer with 0.04% BSA. Cell viability was assessed by Trypan blue exclusion staining and cells counted with a hemocytometer (Countess II, Thermo Fisher Scientific). Cell viability was greater than 95%. Following counting, the samples were diluted to 700 cells/μL. Three replicates were performed since the typical yield of a 10X genomics run is about 2,000 cells, and we wanted to evaluate a higher number, plus we wanted independent biological samples to assess replicability of the data.

### Single-Cell Library Preparation and Sequencing

Libraries were prepared using the Chromium Controller (10X Genomics, Pleasanton, CA, United States) in conjunction with the Single Cell 3’ Reagent Kit v2 kit user guide. Briefly, the cell suspensions were diluted with nuclease-free water according to manufacturer instructions to achieve an estimated cell count of 1,950 to 2,858 per sample. cDNA synthesis, barcoding, and library preparation were then carried out in the Chromium controller according to the manufacturers’ instructions. The libraries were sequenced on an Illumina HiSeq 2500 (Illumina, San Diego, United States) with a read length of 26 bp for read 1 [cell barcode and unique molecule identifier (UMI)], 8 bp i7 index read (sample barcode), and 98 bp for read 2 (actual RNA read). Reads were first sequenced in the rapid run mode, allowing for fine-tuning of sample ratios in the following high-output run. Combining the data from both flow cells yielded approximately 200 million reads per sample.

### Single-Cell Data Analysis

Reads were demultiplexed using Cellranger 2.0.2 (10X Genomics) and mkfastq in conjunction with bcl2fastq 2.17.1.14 (Illumina). The reads were subsequently aligned to the murine reference genome (mm10 with annotations from Ensembl release 84), filtered, and quantified using the Cellranger count command. Cellranger aggr (10X Genomics) was further used to generate an initial secondary analysis (t-distributed stochastic neighbor embedding; t-SNE), principal component analysis (PCA) clustering. Graph-based, as well as K-means (*K* = 2–10) analysis of gene expression was used to identify the 50 most differentially regulated genes that distinguished each PCA cell cluster. Cellranger aggr was used to merge the count matrices from 3 independent samples.

Additional clustering analysis was conducted using R package Seurat (Satija et al., 2015) to merge the data from the three independent samples and generate overall cell clusters. Cells were filtered based on quality control measurements recommended by the Seurat developers. Genes that were expressed in less than 0.1% of cells and cells that expressed less than 750 unique genes were excluded from the analysis. Cells that expressed greater than 7.5% mitochondrial genes were also excluded, as they represent dead or injured cells. After filtering, 6,370 of 6,770 cells remained.

After quality filtering, counts were log normalized. The FindVariableGenes function in Seurat was used to identify 2,207 highly variable genes that were used for downstream analysis. Finally, the data were scaled and subject to PCA to reduce the dimensionality of the dataset.

We used overrepresentation enrichment analysis (ORA) from R package WebGestaltR. Searched geneontology biological process database. Looked for enrichment in significantly differentially expressed genes (adjusted *p*-value < 0.05) for each cluster versus all highly variable genes (*n* = 2,207).

### Identification of Cell Types

Cells were identified by assessing gene expression in the clusters of the three independent samples. Two methods were used to evaluate each of the samples. In the first, the expression of well-recognized cell marker genes was mapped to the graph-based clusters. For a second means of identification, the top 50 differentially expressed genes in each cluster were evaluated for their known expression by different cell types using the GeneCards database and the literature. From both methods, exclusively expressed genes (defined as being expressed by >50% of the cells in a cluster at high levels versus <2.5% of the cells of all other clusters at low levels) that were associated with an individual cell type were then assessed for their known cellular expression in the literature and in the GeneCards database.

### Immunohistochemistry

Expression of select genes used for cell identification was verified by immunohistochemistry. Paraffin sections of MEs were deparaffinized, and antigen retrieval performed with citrate buffer, pH 6.0. The sections were exposed to primary antibodies to: EPCAM (Sino Biological), DYNLRB2 (Thermo Fisher Scientific), COL1A2 (LSBio), LRG (Proteintech), CSFR1 (LSBio) and PTPRCAP (Antibodies.online), DEFB1 (BiossUSA), AREG (LSBio), ECRG4 (Biorbyt), followed by secondary antibodies labeled with Alexa 488 (Abcam).

### Expression of Innate Immune Genes

Acute OM in most children resolves in less than a week even in the absence of antibiotic treatment (Little et al., 2001), a period too short for the full engagement of cognate immunity. This implicates innate immunity in the normal resolution of ME infection. In order to assess the capacity of resting ME cells to rapidly engage innate immunity, we assessed the expression levels of innate immune genes in our single-cell transcriptomes. For this analysis we used the Mouse Genome Informatics gene ontology (GO) list of 1599 innate immune transcripts representing 809 individual genes: GO:0045087.

## Results

### Single-Cell Metrics

The mean number of transcriptomes per sample was 2,257, typical for a single-cell sample on the 10X genomics device, for a total of 6,770 cells. As noted above, quality control eliminating cells with a high proportion of mitochondrial genes likely to be dead or dying cells as well as outliers, left a total of 6,370 cells. A mean of 45.6 million reads/sample resulted in the detection of 17,322 genes for each tissue sample, with an average 20,425 reads/cell, 3,894 UMIs/cell and 1,576 genes/cell. The quality control metrics for each sample are presented in Table 1.

**TABLE 1:** Single cell metrics.

Sample	Reads	Total cells	Genes/cell	Reads/cell	Total genes	UMIs/cell
1	54.0 M	2,858	1,637	18,886	17,719	3,933
2	45.0 M	1,962	1,490	20,920	17,110	3,744
3	41.9 M	1,950	1,600	21,468	17,138	4,005

*Metrics for the three independent samples of ME mucosa, each representing six mouse MEs.*

### Generation of Cell Clusters

**FIGURE 1:**
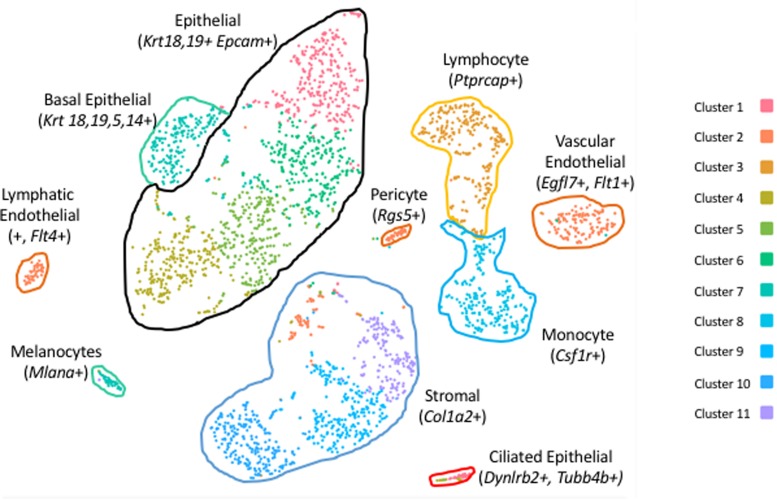
PCA clusters of cells from a sample of six normal mouse ME mucosae, generated by 10X Genomics Cellranger. Eleven separate clusters (1–11) were produced. Marker genes were used to identify the cells of each cluster.

**FIGURE 2:**
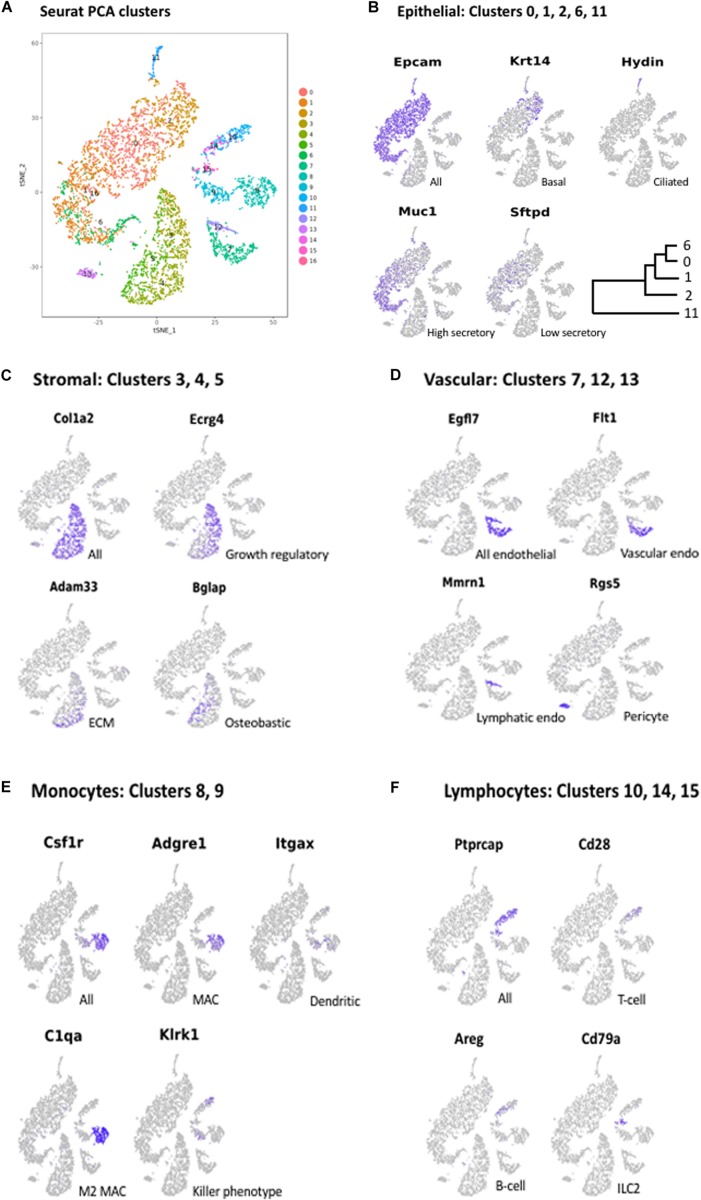
**(A)** PCA clusters generated from the cells of all three mucosal samples, generated with Seurat. Seventeen clusters (0–16) were identified. Marker genes were again used to determine the cells types present in each cluster. Some cell types that were grouped in contiguous clusters by Cellranger were separated in the Seurat analysis. Marker gene expression in: **(B)** Epithelial cell clusters (0, 1, 2, 6, 11); **(C)** Stromal cell clusters (3, 4, 5); **(D)** Vascular cell clusters (7, 12, 13); **(E)** Monocyte clusters (8, 9); and **(F)** Lymphocyte clusters (10, 14, 15).

Clustering of ME cell transcriptomes using the 10X Cellranger pipeline resulted in a similar pattern of cell groupings for each of the samples, identifying 11 (Figure 1), 8 and 7 clusters in the three separate samples. Some of these clusters were subdivisions of spatially contiguous cell groups, and generally it was variation in the number of these subgroups that produced differences in cluster number between samples. However, later analysis confirmed that marker genes present in the sample with the most clusters (11) also distinguished cell groups in the two samples with fewer clusters, even though they had not been separated by PCA. To increase the depth of analysis, cell sequences from the three samples were merged and analyzed using Seurat. Seurat t-SNE clustering of the merged samples yielded 17 cell clusters, which are illustrated in Figure 2A. Several clusters consisted of discrete groups of cells. Others were parts of multi-cluster cell groupings, as observed in the Cellranger analysis.

### Identification of ME Cell Types

As noted above, the expression of cell type marker genes that are well recognized in the literature was used to identify ME cell types (Figure 2). Evaluation of cluster-specific genes (expressed by the majority of cells in a single cluster and very few cells from other clusters) was also used to confirm ME cell type identity, when the literature or the GeneCards database indicated that expression was limited to a single cell type. Genes used to identify ME cell types are presented in Table 2.

**TABLE 2:** Genes used to identify ME cell types.

**Epithelial cells (all)**	*Epcam, Krt18, Krt19*
Basal	*Krt5, Krt14*
Ciliated	*Spag6l, Hydin*
Secretory	*Muc1, Lyz*
**Stromal cells (all)**	*Col1a2*
Endothelial cells	*Egfl7*
Vascular	*Flt4*
Lymphatic	*Flt1*
Pericytes	*Rgs5*
Melanocytes	*Mlana*
**Monocytes (all)**	*Csf1r*
Macrophages	*Adgre1* (F4/80)
M2	*C1qa*
Dendritic cells	*Itgax*
Cytotoxic phenotype	*Klrk1*
**Lymphocytes (all)**	*Ptprcap*
T-cell	*Cd3d*
B-cell	*Cd79a*
Type 2 Lymphoid cell	*Areg*

*These genes were reliable indicators of cell types in the normal ME.*

#### Epithelial Cells

The largest number of cells (51.5% of all cells in the merged samples) showed expression of genes typical of epithelia (Hackett et al., 2011), including the epithelial cell adhesion gene *Epcam* (Figure 2B), the cytokeratin genes *Krt18* and *Krt19*, several claudin genes, and *Muc16*. This included the cells of Seurat Clusters 0, 1, 2, 6, and 11. Two of these clusters were readily identified as epithelial subtypes: Cluster 2 exclusively expressed *Krt5*, *Krt14* (Figure 2B), and *Krt17*, recognized markers of basal epithelial cells. Cluster 11, physically separate from the other epithelial clusters in the Seurat PCA analysis (Figure 2A), exclusively expressed many genes characteristic of ciliated cells (Hoh et al., 2012), including those encoding dynein axosomal heavy (e.g., *Dnah5*) *Intermediate* and light chains, the dynein regulator *Dynlrb2* (dynein light chain roadblock 2) as well as *Hydin* (axonemal central pair apparatus protein) (Figure 2B).

#### Melanocytes

Embedded within the larger epithelial cell grouping that included Clusters 0, 1, 2 and 6 was a small number of cells (Cluster 16, consisting of only 1.8% of all cells) that did not express epithelial markers. The cells of this cluster uniquely expressed markers for melanocytes (Yang et al., 2014), including many involved in melanin synthesis: *Pmel* (premelanosome protein), *Mlana* (melan-A) which is required for PMEL function, *Dct* (dopamine tautomerase, involved in PMEL synthesis), *Slc45a2* (Solute carrier family 45 member 2) involved in melanin synthesis and *Tyrp1* (tyrosine-related protein 1) which regulates melanin synthesis).

#### Stromal Cells

After epithelial cells, the next largest group of cells (22.6% of all cells) expressed genes typical of stromal cells. This included Clusters 3, 4, and 5 (Figure 2A), the cells of which expressed numerous extracellular matrix (ECM)/connective tissue proteins, such as *Col1a1* (collagen type 1 alpha 1), *Col1a2* (Figure 2C), *Fbln1* (fibulin 1, a fibrillar ECM), *Wisp2* (Wnt1 inducible signaling pathway protein, a connective tissue growth factor); *Adamts5* (ADAM metallopeptidase with thrombospondin type 1), a connective tissue organization factor, *Fmod* (fibromodulin) collagen fibrillogenesis factor involved in ECM assembly, and *Cdh11* (cadherin 11), an ECM synthesis regulator.

#### Endothelial Cells and Pericytes

Cells associated with the vasculature comprised 9.2% of all cells on our samples. Clusters 7 and 12 exclusively expressed the endothelial cell marker *Egfl7* (encoding a secreted endothelial protein involved in angiogenesis) (Figure 2D). They were also the only cells expressing several other genes related to angiogenesis including Lrg*1* (A TGF-beta binding protein), *Mmrn2* (A TGF-beta antagonist), *Rasip1*, *Vegfr2*, and *Sox18*. In addition, they expressed *Selp* (P selectin) and *Icam2*, both involved in endothelial recruitment of leukocytes. The cells of Cluster 13 expressed marker genes for pericytes, including *Rgs5* (Figure 2D), a hypoxia-inducible G-protein regulatory subunit involved in angiogenesis and regulation of leukocyte extravasation.

#### Monocytes

Monocyte lineage cells comprised 8.52% of our samples. The cells of Clusters 8 and 9 uniquely expressed many monocyte marker genes, including *Csf1r* (colony stimulating factor 1 receptor) (Figure 2E), *Aif1* (monocyte activation) *c300d* (receptor involved in innate immunity), *Lst1* (lymphocyte proliferation inhibitor), *Clec12a* (negative regulator of monocyte and granulocyte function) and *Ccl2* (macrophage chemoattractant). Cluster 8 cells also exclusively expressed macrophage-specific markers, such as *Adgre1* (F4/80 antigen) (Figure 2E), *Ccl3* (MIP1alpha) and *Mrc1* (macrophage mannose receptor). Many but not all cells in Cluster 9 preferentially expressed *Itgax* (mature dendritic cell marker) (Figure 2E) and *Cd209a* (dendritic cell adhesion molecule), consistent with dendritic cell identity. The non-macrophage, non-dendritic cell monocytes were a mixed population of inflammatory (*Ccr2*^+^, *Ly6C2*^+^) and resident, homeostatic (*Cx3cr1*^+^, *Ly6C2*^–^) phenotypes (Gordon and Taylor, 2005).

#### Lymphocytes

**FIGURE 3:**
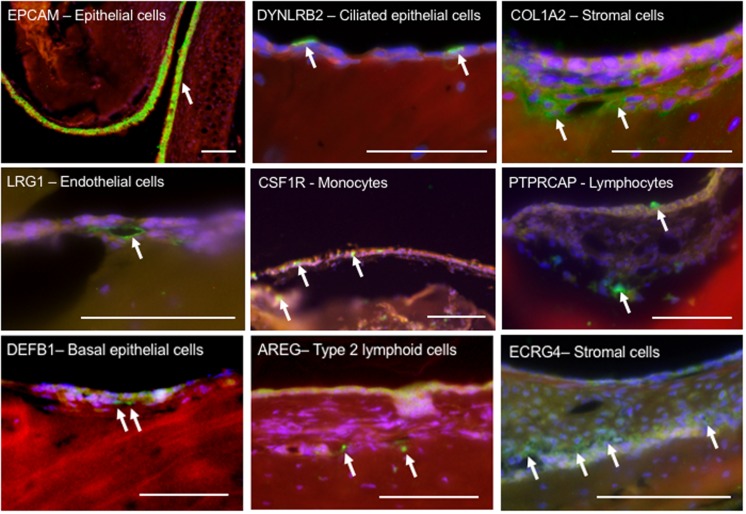
Immunohistochemical localization of select proteins encoded by marker genes, used to identify cell types in the ME mucosa. Paraffin sections were labeled with phalloidin (red), DAPI (blue) and Alexa 488-conjugated secondary antibodies (green). The upper six panels represent marker genes used to identify cell types known to be present in the ME. The three lower panels represent less expected findings: DEFB1 expression in basal epithelial cells; AREG indicating type II lymphoid cells; and ECRG4, often expressed in epithelia, in stromal cells. Scale bars represents 100 μm.

The lymphocytes of Clusters 10, 14, and 15 (comprised 6.2% of our samples. All exclusively expressed a murine lymphocyte-specific gene *Ptprcap* (encoding a key regulator of lymphocyte activation) (Figure 2F). Cluster 10 cells also expressed *Cd2* and *Cd28* (Figure 2F), markers of both T- and NK-cells. The cells of Cluster 14 did not express specific T-cell or B-cell markers. However, they exclusively expressed *Areg* (Figures 2F, 3), an immunoregulatory member of the EGF family which is produced by type 2 innate lymphoid cells (ILC2s) (Zaiss et al., 2015). Consistent with an ILC2 phenotype, they also expressed *Il7r* and *Thy1*, *Il13*, and *Gata3* (Gasteiger et al., 2017). Cluster 15 cells expressed B-cell markers including *Cd19*, *Cd79a* (Figure 2F) and *Cd79b* (involved in B-cell antigen recognition), *H2-dmb2* (a B-cell class II molecule), *Mzb1* (which regulates Ca^2+^ stores to diversify B-cell function).

The expression of selected genes used to identify ME cell types is further illustrated by immunohistochemical labeling in Figure 3.

### Differential Expression of Genes by ME Cell Clusters

**FIGURE 4:**
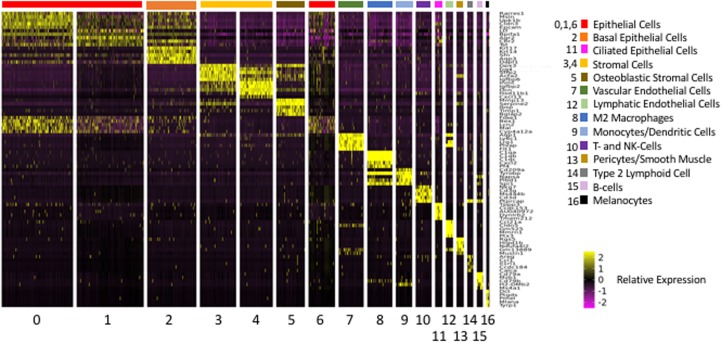
Heatmap illustrating the 5 most differentially regulated genes for each of the 17 Seurat cell clusters. Most clusters were clearly delineated by the differential gene expression. However, three epithelial clusters (0, 1, and 6) showed somewhat overlapping patterns of expression.

The above analysis employed marker genes to identify cell types in our ME samples. However, the majority of the genes that were significantly differentially expressed between clusters, and which defined them in PCA analysis, were not markers for a given cell type. A total of 2,207 genes were differentially expressed between Seurat t-SNE clusters. For purposes of illustration, Figure 4 shows a heat map of the top 5 genes that were differentially regulated between each of the various clusters, and that contributed to cluster generation. It can be seen from the figure that most clusters expressed gene sets that clearly differentiated them from other cell groups. Genes that were highly expressed by different ME cell types, either exclusive to that type (as defined previously) or strongly preferentially, are described below.

#### Epithelial Cells

Many of the genes exclusively expressed by cells in all five ME epithelial populations are involved in fluid or solute regulation, including *Aqp5*, *Cldns 3, 4, and 7*, *Fxyd3*, *Kcnj16*; *Atp1b1*, *Fxyd3* and *Slc6a1*.

The cells of Clusters 0, 1, and 6 appeared to be closely related and expressed relatively few genes exclusively. Rather, this group of clusters expressed genes in common, often exhibiting relative differences in expression. A number of genes were expressed in these clusters in a gradient, with the descending order 1, 6 and 0. This included *Muc1* (Figure 2B), *Muc5b*, *Muc16*, *Tff2* (Trefoil factor 2, mucin stabilization gene), *Reg3g* (secreted lectin), as well as *Lyz2* (lysozyme) and *Ltf* (lactotransferrin), both antimicrobials. Also expressed with this gradient were *Agr2* (anterior gradient 2) involved in mucin assembly and *Mgst1* (microsomal glutathione S-transferase 1) involved in leukotriene and prostaglandin synthesis. These gene gradients are consistent with Cluster 1 cells exhibiting the highest level of secretory activity. Cluster 1 cells also expressed high levels of *Upk1b*, involved in the stabilization and strengthening of apical cell membranes, suggesting presence at the luminal surface of the mucosa.

Genes expressed with an opposite gradient, highest in Cluster 0 and lowest in Cluster 1, included *Bcam* and *Igfbp5*, involved in ECM binding, and *Sftpd* (Figure 2B) encoding surfactant protein D. Genes that defined Cluster 6 included *Cyp4a12a* (iron binding oxyreductase), *Mal* (vesicle trafficking from Golgi), *Lcn2* (iron sequestration), *Bex1* (growth factor signaling) and *Foxe1* (transcription factor involved in TGF and WNT regulation). However, these genes were also expressed in Clusters 0, at higher levels than in Cluster 6, and to a lesser extent in Clusters 1 and 2 (see Figure 4).

The basal epithelial cells of Cluster 2 cells strongly expressed *Sfn* (a regulator of cell signaling and cell cycle), *Aqp3*, *Dapl1* (G coupled receptor activity), and *Anax8.* Aquaporin 3 is found in the basolateral membranes of kidney collecting duct cells, where it provides a pathway for water to exit these cells. This may play a similar role in the exit of water from the ME mucosal epithelium at its basal surface. Annexin 8 is a calcium binding protein found in mature and functional epithelial cells, where it participates in exocytosis. Interestingly, basal epithelial cells were also the only ME cells to strongly express *Defb1*, encoding the antimicrobial, beta defensin 1 (Figure 3).

In addition to many genes related to cilia, the ciliated epithelial cells of Cluster 11 exclusively expressed *Muc4*, encoding a membrane mucin that can activate ERBB2 and stimulate epithelial proliferation. They also preferentially expressed an unusually high number of genes for which no function has been well described, including among others *Tmem212*, *Ccdc153*, *C9orf116*, *Fam183b*, *Sec14l3*, and antisense IncRNA *AU40972*.

#### Stromal Cells

Cluster 3 was distinguished from other clusters by significantly higher levels of expression of growth regulators, including *Ecrg4* (epithelial cell growth regulator) (Figures 2C, 3) which in other tissues is often expressed in epithelial cells (Kurabi et al., 2013), *Sfrp2* (Wnt signaling modulator), *Serpinf1* (angiogenesis and cell differentiation inhibitor) and *Aspn* (TGFß and BMP inhibitor).

Cluster 4 exclusively expressed *Adam33* (cell−ECM interactions) (Figure 2C), *Dpt* (ECM assembly) and *Col28a1* (collagen chain trimerization). The cluster preferentially expressed other genes related to ECM generation: including *Gpc6* (growth factor and ECM receptor), *Dcn* (ECM assembly), *Mfap4* and *Mfap5* (ECM proteins); as well as genes involved in defense against infection: *Cxcl13* (anti-microbial, B-cell chemoattractant) and *Adm* (antimicrobial and fluid regulation).

The cells of Cluster 5 strongly expressed *Mmp13* (ECM breakdown), *Serpine2* (regulator of cell signaling) and *Timp1* (MMP inhibitor). They also exclusively expressed several genes consistent with osteoblast function, including *Bglap* (an abundant Ca-binding bone protein) (Figure 2C), *Bglap2* (a hormone secreted by osteoblasts), *Ibsp* (a major structural protein in bone) and *Sfrp4* (involved in bone morphogenesis), as well as *Tnc* (ECM protein tenascin C), *Podnl1* (collagen binding), and *Ackr4* (a decoy receptor that inactivates cytokines).

#### Endothelial Cells

Of the two groups of endothelial cells, Cluster 7 strongly expressed *Aqp1*, *Ly6c1* (an immunocyte antigen also expressed by endothelial cells), *Lrg1* (TGFß receptor binding) and *Plvap* (microvascular permeability). They exclusively expressed *Flt1* (VEGFR1) (Figure 2D), *Aplnr* and *Vwf (Von Willebrand factor)*, consistent with vascular endothelium, as well as the scavenger cytokine receptor *Ackr1*. Cluster 12 cells exclusively expressed *Prox1 (a homeobox protein)*, *Flt4 (VEGF receptor 3)* and *Reln (Reelin)*, all markers for lymphatic endothelial cells, as well as *Ccl21a* (T-cell chemotaxis), *Cldn5* (tight junction protein), *Gm525* (unknown function), *Ptx3* (angiogenesis and inflammation), *Mmrn1* (a factor V/Va receptor) (Figure 2D) and the cytokine scavenger receptor gene *Ackr2*.

#### Pericytes

The pericytes of Cluster 13 exclusively expressed three hypoxia-inducible mitochondrial genes involved in regulating the shift between glucose and glycogen metabolism: *Higd1b*; *Ndufa4l2* and *Cox4i2*. They were the only ME cells to express genes involved in regulating vascular tone, including *Des*, *Olf558*, *Myh11*, *Myocd* and *Kcne4* (Jepps et al., 2015). They also expressed *Cspg4*, encoding a proteoglycan that stimulates endothelial cell motility during microvascular morphogenesis and *Ephx3*, encoding a protein involved in water permeability barriers. They strongly expressed *GM13889* (unknown function) and *Mustn1* (muscle development).

#### Macrophages

The macrophages of Cluster 8 preferentially expressed genes observed in M2 (alternatively activated) macrophages, such as those encoding the complement components *C1qa* (Figure 2E), *C1qb* and *Ciqc* and the fractalkine receptor *Cx3cr1* (Italiani and Boraschi, 2014). They also strongly expressed *Cxcl2* (macrophage inflammatory protein 2), *Pf4* (neutrophil and monocyte chemotaxis) *Tyrobp* (neutrophil activation), *Plbd1* (hydrolase activity) and *Spi1* (macrophage differentiation). Interestingly, none of the cells in this clusters expressed the mature macrophage marker *Itgam* (MAC-1), but many expressed *Cd33*, characteristic of immature macrophages.

#### Dendritic Cells/Monocytes

Most of the cells in Cluster 9 preferentially expressed *Itgax* (mature dendritic cell marker) and *Cd209a* (dendritic cell adhesion molecule), consistent with dendritic cell identity. However, these cells also expressed the NK gene *Klrk1* (Figure 2E). This indicates that they are primarily NK dendritic cells, which possess cytotoxic capability. These cells are usually present as a small subset of dendritic cells in blood and tissue (Chan et al., 2006), but appear to represent about 40% of dendritic cells (*Itgax*^+^) in the ME. In common with Cluster 8, the dendritic cells and monocytes of Cluster 9 strongly expressed *Tyrobp*, *Plbd1* and *Spi*. They also exclusively expressed *Cd209a* (pathogen receptor). As noted above, less differentiated monocytes expressed genes consistent with either the classical (pro-inflammatory) or resident (homeostatic) phenotypes.

#### Lymphocytes

Many of the lymphocytes of Clusters 10 expressed the T-cell receptor genes *Cd3d* and *Cd3g*, as well as *CD3e* and *Cd3z*, indicating that they are gamma/delta T-cells. Consistent with this identity, very few expressed *Cd4*. Gamma/delta T-cells are common in mucosal and other barrier tissues where they serve as part of the front-line defense against infection. Many also expressed *Nkg7* (a natural killer cell granule protein) suggesting a cytotoxic phenotype. A small subpopulation expressed *Cd8a* and *Cd8b1*. *Cd8ab*-positive gamma-delta T-cells have been identified as a unique cytotoxic population that is negatively correlated with disease states (Kadivar et al., 2016). The larger population of Cd8^–^/Cd4^–^ cells are known as double-negative T-cells. In other tissues, the majority of double-negative T-cells bear the alpha/beta receptors consistent with cognate immunity (Antonelli et al., 2006). However, in the ME mucosa the majority appear to bear the gamma/delta receptor more consistent with innate immune function. Gamma/delta T-cells are also involved in M2 macrophage polarization (Mathews et al., 2015). Cluster 10 cells also strongly expressed *Ms4a4b*, a negative regulator of T-cell proliferation.

The ILC2 cells of Cluster 14 type exclusively expressed *Il5* and *Il13*, Th2 cytokines associated with this cell type, as well as *Il1rl1* and *Icos*, which have been associated with helper cell function, and *Cxcr6*, associated with memory, naïve (*Cd28*^+^) and regulatory (*Il7r*^+^) T-cells. They also strongly expressed, *Il1rl1* (possibly helper T-cell function), *Ccdc184* (unknown function), and Calca (calcium regulation, antimicrobial).

The B-cells of Cluster 15 expressed many genes related to B-cell function, including *Cd22* (essential for B-cell−B-cell interactions); *Dank1* (mobilization of intracellular B-cell stores); *Spi6* (B-cell development); *Pou2af1* (essential for B-cell response to antigens), *Ms4a1* (B-cell development) and various Fc receptor genes involved in B-cell activation. They also expressed *Pax5*, involved in early but not late B-cell development, suggesting that ME B-cells are not fully mature. A small number of cluster cells expressed *Jchain*, involved in the production of secretory factor.

In addition to evaluating differentially expressed genes, we applied an analysis of the GO: biological processes the genes for which were most highly expressed by different cell clusters. Not surprisingly the results summarized in Table 3, are largely consistent with functions that can be inferred from the differentially regulated genes presented above.

**TABLE 3:** Single-cell cluster enrichment of GO: biological process.

GO category	*P*-value
**Cluster 0 (Epithelial cells)**	
Negative regulation of cell motility	2.0631e−7
Negative regulation of cell migration	9.0561e−7
Extracellular matrix organization	8.8733e−8
Negative regulation of cellular component movement	3.3807e−7
Negative regulation of locomotion	6.5930e−7
**Cluster 1 (Epithelial cells)**	
Extracellular matrix assembly	0.0000088218
Collagen metabolic process	0.0000034043
Extracellular matrix organization	4.0817e−9
Extracellular structure organization	2.7391e−8
Cell growth	0.0000029102
**Cluster 6 (Epithelial cells)**	
Protein trimerization	0.00018877
Cellular response to amino acid stimulus	0.000026320
Cellular response to acid chemical	0.000072229
Lung development	0.00018877
Respiratory tube development	0.00022526
**Cluster 2 (Basal epithelial cells)**	
Collagen biosynthetic process	0.000040927
Collagen metabolic process	0.00018409
Protein localization to plasma membrane	0.000059560
Bone mineralization	0.000055506
Transforming growth factor beta receptor signaling pathway	0.000045449
**Cluster 11 (Ciliated epithelial cells)**	
Axoneme assembly	0
Cilium movement	0
Cilium or flagellum-dependent cell motility	1.9154e−10
Cilium-dependent cell motility	1.9154e−10
Axonemal dynein complex assembly	1.9154e−10
**Cluster 3 (Stromal cells)**	
Collagen fibril organization	0.0000091862
Collagen metabolic process	0.0000037847
Protein processing	0.000068852
Extracellular matrix organization	4.5168e−9
Extracellular structure organization	7.6653e−8
**Cluster 4 (Stromal cells)**	
Collagen fibril organization	0.0000043023
Embryonic skeletal system development	0.000014268
Negative regulation of cellular response to growth factor stimulus	0.0000039117
Collagen metabolic process	0.0000068214
Extracellular matrix organization	3.7315e−11
**Cluster 5 (Stromal cells)**	
Collagen fibril organization	1.5268e−9
Regulation of bone mineralization	5.1255e−8
Regulation of biomineral tissue development 6.3049e−9	
Bone mineralization	7.6798e−10
Biomineral tissue development	1.7298e−11
**Cluster 7 (Vascular endothelial cells)**	
Negative regulation of cellular component movement	2.5616e−8
Regulation of plasma membrane bounded cell projection	4.2233e−8
Regulation of cell projection organization	5.2664e−8
Angiogenesis	6.4595e−11
Blood vessel morphogenesis	9.2663e−11
**Cluster 12 (Lymphatic endothelial cells)**	
Endothelial cell differentiation	0.0000057055
Endothelium development	0.0000080317
Sprouting angiogenesis	0.000010961
Negative regulation of angiogenesis	0.0000091829
Negative regulation of blood vessel morphogenesis	0.000013967
**Cluster 13 (Pericytes)**	
Membrane repolarization	0.000084352
Actin-mediated cell contraction	0.0000096969
Actin filament-based movement	0.000018559
Notch signaling pathway	0.0000090719
Regulation of heart contraction	0.00017208
**Cluster 8 (Macrophages)**	
Tumor necrosis factor production	5.0342e−8
Regulation of tumor necrosis factor production	3.3417e−8
Cellular response to molecule of bacterial origin	4.9622e−8
Response to molecule of bacterial origin	2.1447e−8
Response to organonitrogen compound	3.3417e−8
**Cluster 9 (Monocytes, Dendritic cells)**	
Cellular response to radiation	0.000033926
Activation of innate immune response	0.000071442
Tumor necrosis factor production	0.000017344
Regulation of tumor necrosis factor superfamily cytokine production	0.000043714
Tumor necrosis factor superfamily cytokine production	0.000043714
**Cluster 10 (T-cells, NK cells)**	
T-cell receptor signaling pathway	5.3595e−7
Positive regulation of leukocyte cell-adhesion	3.9513e−7
Positive regulation of lymphocyte activation	2.3319e−7
T-cell differentiation	9.7306e−8
T-cell activation	4.4670e−12
**Cluster 14 (ILC2s)**	
Positive regulation of leukocyte differentiation	0.0000018086
Regulation of lymphocyte differentiation	0.0000011529
Positive regulation of hemopoiesis	0.0000013987
Positive regulation of lymphocyte activation	0.000002159
Regulation of hemopoiesis	1.1023e−7
**Cluster 15 (B-cells)**	
B cell receptor signaling pathway	2.9347e−11
Regulation of B cell proliferation	7.1741e−8
B cell proliferation	3.0189e−8
B cell activation	6.8834e−15
Antigen receptor-mediated signaling pathway	2.8936e−9
**Cluster 16 (Melanocytes)**	
Sister chromatid segregation	8.2230e−10
DNA packaging	2.8980e−9
DNA conformation change	5.2742e−10
Chromosome segregation	9.2013e−11
Nuclear chromosome segregation	1.4193e−9

*The five GO categories with the highest enrichment ratios are listed for each single-cell cluster.*

### Expression of Innate Immune Genes by ME Cell Types

The genes reviewed above were identified based purely on differential expression across cell clusters. In order to evaluate the expression of genes specifically related to OM, we first assessed genes related to innate immunity across ME cell clusters and types. Acute OM in the average child resolves in less than one week (Little et al., 2001). This is not sufficient time to mount a robust cognate immune response, which implies that normal resolution of acute OM is mediated by innate immunity. Indeed, studies in mice with deletions of individual genes for innate immune receptors or effectors have found that, while lack of some genes leads to more severe deficits in recovery from bacterial OM, virtually all show a deficit (Kurabi et al., 2016). This finding indicates that the innate immune system participates in normal OM recovery, and that the genetic machinery with which to initiate innate immunity resides in the cells of the normal ME. We therefore evaluated the expression of genes in Mouse Genome Informatics GO category 0045087 “Innate Immunity.” Out of 809 genes in the category, expression of 805 was detected in cells of the normal ME.

For a few genes, there was broad expression across all ME cell types. These included *Bipfa1* (contributes to airway surface liquid homeostasis and proper clearance of mucus) as well as three ribosomal proteins with innate immune subfunctions: *Rpl13a* (suppression of inflammatory genes); Rpl39 (viral gene transcription) and Rps19 (suppression of interferon production).

**FIGURE 5:**
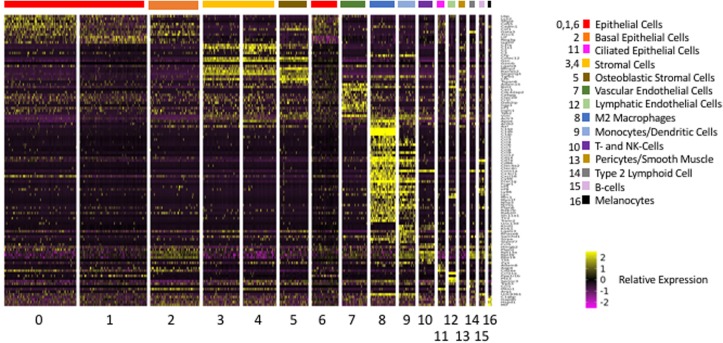
Heatmap illustrating the expression of the 109 most differentially regulated innate immune genes, arranged by cell cluster in which they are differentially expressed. Cluster 8 cells (macrophages) expressed the greatest number.

For a total of 520 innate immune genes, there were significant expression differences between cell types. A heat map illustrating the expression of the 520 genes across all ME cells is presented in Supplementary Figure S1, while Figure 5 shows the expression of a more readily visualized set of 109 innate immune genes, those with the most robust expression, ordered by cell type. Table 4 lists the most differentially expressed innate immune genes for each cell cluster. All clusters expressed distinct sets of innate immune genes.

**TABLE 4:** Single-cell Cluster Expression of Innate Immune Genes.

Gene	Protein	Innate immune function
**Cluster 0 (Epithelial cells)**		
*Lbp*	LPS Binding Protein	Endotoxin receptor component
*Hmgb2*	High mobility group box 2	Innate immune DNA/RNA receptor
*Csf1*	Colony stimulating factor 1	Macrophage differentiation
*Cxcl16*	Chemokine CXCL16	T-cell recruitment
*Ptpn2*	PTP2	T-cell activitation, tissue growth
*Cadm1*	CADM1	NK and T-cell regulation by other cell types
*Cd24a*	CD24A	B-cell differentiation, tissue growth
*Lcn2*	Lipocalin2	Antibacterial
*Sftpd*	surfactant D	Antibacterial, surfactant
*Xrcc5*	DNA repair XRCC5	Antiviral
*Cd55*	DAF	Negative complement regulator
**Cluster 1 (Epithelial cells)**		
*Cd25*	IL2 receptor α	Control of regulatory T-cell, tissue growth
*Ppp1r1b*	PPP1R1B	Regulatory T-cell activation, cell survival
*Ltf*	Lactoferrin	Antimicrobial
*Reg3*γ	REG3γ	Antimicrobial
*Isg20*	ISG20	IFN-stimulated antiviral
**Cluster 6 (Epithelial cells)**		
*Sftpd*	surfactant D	antibacterial, surfactant
**Cluster 2 (Basal epithelial cells)**		
*Lbp*	LPS Binding	endotoxin receptor component
*Hmgb2*	High mobility group box 2	innate immune DNA/RNA receptor
*Cadm1*	CADM1	NK and T-cell regulation by other cell types
*Gata3*	GATA3	Immune cell differentiation, tissue growth
*Rab20*	RAB20	endocytosis, antibacterial responses
*Xrcc5*	DNA repair XRCC5	antiviral response
*Ifitm1*	IFITM1	Inhibition of viral entry
*Rpl39*	Ribosomal protein L39	Viral gene transcription
*Rpl13a*	Ribosomal	Suppression of inflammation
*Rps19*	Ribosomal	Suppression of interferon
*Axl*	AXL receptor tyrosine kinase	TLR inhibition
*Cd55*	DAF	negative complement regulator
**Cluster 11 (Ciliated epithelial cells)**		
*Adam8*	ADAM8	leukocyte migration
*Cd24a*	CD24A	B-cell differentiation, tissue growth
*Ifitm1*	IFITM1	inhibition of viral entry
*Aqp4*	Aquaporin4	water permeability, response to inflammation
**Clusters 3, 4, 5 (All stromal cells)**		
*Gsn*	Gelsolin	TLR endocytosis
*Rarres2*	Chemerin	Chemotactic, anti-inflammatory
*C1ra*	Complement C1 component	Complement response
*Colec12*	Collectin-12	Antimicrobial responses
*Serping1*	Serpin G1	Negative complement regulator
*Axl*	AXL receptor tyrosine kinase	TLR inhibition
**Cluster 3 (Stromal cells)**		
*C1s1*	Complement C1 component	Complement response
*Cr1l*	C1 receptor like	Complement activation
*Samhd1*	SAMHD1	Mediates TNF inflammatory responses
*Lgals9*	Galactin9	Negative regulation of T- and NK-cells
*Ifitm1*	IFITM1	Inhibition of viral entry
*Clec2d*	CLEC2D	Protection against NK cell lysis
**Cluster 4 (Stromal cells)**		
*C1s1*	Complement C1 component	Complement response
*C2*	Complement C2	Complement response
*C3*	Complement C3	Complement response
*Apoe*	Apolipoprotein E	Leukocyte regulation, lipid metabolism
*Zyx*	Zyxin	Viral pathogen receptor signaling
*Bst2*	Tetherin	Antiviral
*Serinc3*	Serine incorporator 3	Antiviral
*Trp53*	P53	Antiviral
*Ifitm1*	IFITM1	Inhibition of viral entry
*Lgals9*	Galectin 9	Negative regulator of T- and NK-cells
**Cluster 5 (Stromal cells)**		
*Lgals3*	Galectin 3	Acute inflammation activator
*Csf1*	Colony stimulating factor 1	Macrophage differentiation
*Trp53*	P53	Antiviral
*Tgfb1*	TGFβ1	Multifunctional, immunocyte inhibition
*Tspan6*	Tetraspanin 6	Negative innate immune regulator
**Cluster 7 (Vascular endothelial cells)**		
*Vim*	Vimentin	Viral and bacterial attachment
*Samhd1*	SAMHD1	Mediates TNF inflammatory responses
*Dab2ip*	DAB2 interacting protein	TNF, IFN and LPS signaling pathways
*Irf1*	IFN response factor	IFN production
*Adam15*	ADAM15	Epithelial−T-cell interaction
*Cav1*	Caveolin 1	T-cell activation, TGFß1 inhibition
*Iigp1*	IFN-inducible G Protein 1	IFN-inducible antimicrobial
*Cebpg*	CEBPG	IL4 gene activation
*Irgm1*	IRGM1	Mucosal immune response inhibition
**Cluster 12 (Lymphatic endothelial cells)**		
*Mrc1*	Mannose receptor C1	Pathogen neutralization
*Ptx3*	Pentraxin 3	Positive regulation of innate immunity
*Ccl21*	Chemokine CCL21	T-cell chemotaxis
*Arrb2*	Arrestin β2	Cytokine/chemokine signaling pathways
*Bst2*	Tetherin	Anti-viral
*Serinc3*	Serine incorporator 3	Antiviral
*Tspan6*	Tetraspanin 6	Negative innate immune regulator
**Cluster 13 (Pericytes)**		
*C1s1*	Complement C1 component	Complement response
*Cav1*	Caveolin 1	T-cell activation, TGFß1 inhibition
*Ifitm1*	IFITM1	Inhibition of viral entry
**Cluster 8 (Macrophages)**		
*Tlr2*	Toll-like receptor 2	Pathogen receptor
*Cd14*	CD14	LPS receptor component
*Ly86*	MD-1	LPS receptor component
*Nlrp3*	NALP3	Pathogen receptor, inflammasome
*MyD88*	MYD88	TLR signaling adaptor
*Mrc1*	Mannose receptor C1	Pathogen neutralization
*Unc93b1*	UNC93B1	Required for TLR DNA recognition
*Cfp*	Complement factor P	Alternative complement response
*C1qa,b,c*	Three C1q A-chains	Complement response
*Irf5, Irf8*	IFN response factors	IFN production
*Cd86*	CD86	T-cell activation, IL2 production
*Actr3*	ACTR3	Phagocytosis
*Trem2*	TREM2	Immune activation in phagocytes
*Cd74*	CD74	MHC class II antigen processing
*Clec4a2*	Lectin-like immunoreceptor	Antigen presentation
*Apoe*	Apolipoprotein E	Macrophage, T- and NK-cell regulation
*Arrb2*	Arrestin β2	Cytokine/chemokine signaling pathways
*Ccl2*	Chemokine CCL2	Macrophage chemotaxis
*Ccl3*	Chemokine CCL3, MIP1α	Monocyte, PMN chemotaxis
*Ccl4*	Chemokine CCL4, MIP1β	NK cell, monocyte chemotaxis
*Ccl5*	Chemokine CCL5, RANTES	T-cell chemotaxis
*Ccl9*	Chemokine CCL9, MRP2	Dendritic cell chemotaxis
*Cxcl16*	Chemokine CXCL16	T-cell, NK-cell chemotaxis
*Coro1a*	Coronin 1A	Lytic granule secretion
*Cx3cr1*	Fractalkine receptor	T-cell, monocyte chemotaxis
*Fcer1g*	FCER1γ	IgE receptor component
*Lyn*	LYN	Diverse immune signaling pathways
*Nrros*	Negative regulator of ROS	TGFβ1 activation in macrophages
*Ptpn6*	PTPN6	Hematopoietic cell signaling
*Rab20*	RAB20	Endocytosis, antibacterial responses
*Slc11a1*	SLC11A1	Iron transporter, antibacterial
*Bst2*	Tetherin	Anti-viral
*Sirpa*	SIRPA	Dendritic cell activation inhibitor
*Tgfb1*	TGFβ1	Multifunctional, immunocyte inhibition
*Axl*	AXL receptor tyrosine kinase	TLR inhibition
*Rab7b*	RAB7B	Negative regulation of TLRs
**Cluster 9 (Monocytes, Dendritic cells)**		
Cluster 9 cells expressed a subset of genes expressed by the macrophages of Cluster 8: *Cfp, Tgfb1, Vim, Actr3, Ccl6, Ccl9, Cd74, Coro1a, Cybb, Fcer1g, Ly86, Nrros, Ptpn6* and *Unc93b1*. However, they also expressed:		
*Myo1f*	Myosin 1F	Immune cell motility
*Klrd1*	KLRD1	MHC recognition by cytotoxic cells
*Klrk1*	KIRK1	Cytotoxicity of virus-infected cells
*Slamf7*	SLAMF7	NK cell activation
*Lgals3*	Galectin 3	Monocyte, macrophage chemotaxis
*Rnase6*	RNASE6	Antibacterial
*Rpl39*	Ribosomal protein L39	Viral gene translation
*Rps19*	Ribosomal protein S19	Viral gene translation
**Cluster 10 (T-cells, NK cells)**		
*Hmgb2*	HMGB2	Innate immune DNA/RNA receptor
*Txk*	TXK tyrosine kinase	Th1 cytokine production
*Coro1a*	Coronin 1A	Lytic granule secretion
*Klrd1*	KLRD1	MHC recognition by cytotoxic cells
*Klrk1*	KIRK1	Cytotoxicity of virus-infected cells
*Ccl5*	Chemokine CCL5, RANTES	T-cell chemotaxis
*Rpl14a*	Ribosomal protein 14a	Viral replication and gene translation
**Cluster 14 (ILC2s)**		
*Coro1a*	Coronin 1A	Lytic granule secretion
*Samhd1*	SAMHD1	Mediates TNF inflammatory responses
*Arg1*	Arginase 1	Promotes acute type 2 inflammation
*C1qbp*	C1q binding protein	Multiple immune/inflammatory responses
*Ccl1*	Chemokine CCL1	Monocyte chemotaxis
**Cluster 15 (B-cells)**		
*Ly86*	MD-1	LPS receptor component
*Unc93b1*	UNC93B1	Required for TLR DNA recognition
*Cd74*	CD74	MHC class II antigen processing
*Ptpn6*	PTPN6	
*Coro1a*	Coronin 1A	Lytic granule secretion
*Slamf7*	SLAMF7	NK cell activation
*Irf8*	IFN response factor 8	Regulates IFN responses
**Cluster 16 (Melanocytes)**		
*Vim*	Vimentin	Viral and bacterial attachment
*Mif*	MMIF	Pro-inflammatory mediator
*Gapdh*	GAPDH	Inhibits IFN-induced gene expression

The epithelial cells of Cluster 0 expressed *Lbp* (component of the endotoxin receptor), *Lcl2* (antibacterial protein) *Sftpd* (surfactant protein D), *Xrcc5* (antiviral response) *Cd55* (negative complement regulator) and *Hmgb2* (innate immune DNA/RNA receptor). They also expressed genes related to leukocyte recruitment and activation, including *Csf1* (macrophage differentiation) and *Cxcl16* (T-cell recruitment). Cluster 1 epithelial cells expressed the genes for the anti-microbials lactoferrin and REG3γ, and the interferon-stimulated antivirals CD25 and ISG20. The basal epithelial cells of Cluster 2 expressed several complement genes, as well as *Axl* (TLR inhibition), *Rab20* (antibacterial response), and the anti-viral gene *Ifit1*. Cluster 6 epithelial cells expressed *Sftpd*. The ciliated epithelial cells of Cluster 11 expressed a small set of diverse innate immune genes including *Hist1b2bc* (antibacterial responses), *Il1rn1* (receptor-blocking inhibitor of IL1ß), *Ifit1*, and *Aqp4*.

Innate immune genes expressed by all stromal cells of Clusters 3, 4 and 5 included *Colec12*, (antimicrobial response), *Mmp2* (positive regulator of inflammatory NFκB signaling), *Rarres2* (chemotactic and anti-inflammatory factor), and *Serpinb1* (negative complement regulation). Clusters 3 and 4 uniquely expressed several complement factors and *Lgals9* (soluble, negative regulator of T- and NK-cells). Cluster 4 expressed the complement factor gene *C2*. Clusters 3 and 5 expressed *Cfn* (negative complement regulator), while Cluster 5 expressed *Tgfb1* (multifunctional, including inhibition of T-cell Th1, Th2 and cytotoxic phenotypes) and *Tspan6* (negative regulator of innate immunity).

The vascular endothelial cells of Clusters 7 expressed *Adam15* (epithelial−T-cell interactions) *Cav1* (T-cell activation, negative regulation of TGFß1), *Cebpg* (IL4 gene activation), *Dab2ip* (participates in TNF, IFN and LPS signaling pathways), *Iigp1* (IFN-inducible antimicrobial), *Irf1* (IFN production), *Irgm1* (IFN-induced negative regulator of mucosal immune responses), *Vim* (viral and bacterial attachment) and *Samhd1* (response to virus, mediation of TNF response).

The lymphatic endothelial cells of Cluster 12 strongly expressed *Tspan6*, *Mrc1*, *Ccl21a* (all described above) as well as *Bst2* (antiviral), *Arrb2* (involved in multiple signaling pathways including that of CCL19), *Ptx3* (positive regulation of innate response to pathogens) and *Serinc3* (resistance to viral infection).

The pericytes of Cluster 13 expressed the complement gene *C1s1*, *Cav1* (negative regulator of inflammation) and *Ifitm1*.

Some of the innate immune genes expressed by Cluster 8 macrophages are marker genes for this cell types, as noted above. Also expressed preferentially by this cell type were nine genes related to pathogen receptor signaling, a total of ten chemotactic chemokine genes, four genes related to complement response, six immune modulators including four negative regulators and the antibacterial gene *Scl11a1.*

Cluster 9 monocytes and dendritic cells preferentially expressed a subset of the genes expressed by Cluster 8 macrophages, but also expressed two genes encoding NK cell lectins related to cytotoxicity, *Klrd1* and *Klrk1*; *Lgals3* (PMN and mast cell activation, macrophage chemotaxis); *Rnase6* (antibacterial); *Samhd1* (antiviral responses); *Sirpa* (negative regulation of dendritic cells and phagocytosis); Slamf7 (killer cell activation); and *Unc93b1* (intracellular TLR transport).

The T-cells and NK cells of cluster 10 strongly expressed *Coro1a (required for lytic granule secretion from cytotoxic cells)*, *Klrd1*, *Klrk1*, *Ccl5* (leukocyte chemoattractant or chemotaxis inhibitor, depending on splicing), *Hmgb2* (innate immune DNA/RNA receptor), *Rpl14a* (ribosomal protein upregulated by endotoxin, Ceppi et al., 2009) and *Txk* (involved in Th1 cytokine production). The ILC2s of Cluster 14 expressed *Cor1a*, *Samhd1*, *Ccl1* (monocyte chemotaxis), *Arg1* (promotes acute type 2 inflammation), and *C1qbp* (involved in multiple infection and inflammatory responses). The B cells of Cluster 15 expressed *Cd74* (MIF receptor), *Corol1a*, *Irf8* (regulates IFN responses), *Ly86* (involved in endotoxin response with TLR4), *Ptpn6* (hematopoietic cell signaling), *Slamf7* (NK cell activation) and *Unc93b1*.

The melanocytes of Cluster 16 strongly expressed *Vim*, *Gapdh* (in innate immunity, IFNg-induced transcript-selective translation inhibition) and *Mif* (regulation of macrophage function).

## Discussion

The results of this study provide, for the first time, a molecular landscape of the cells that make up the normal mucosal lining of the ME prior to OM. They also identify the resting ME cells that express major determinants of innate immunity. As noted above, innate immunity is responsible for the normal resolution of OM (e.g., Underwood and Bakaletz, 2011; Kurabi et al., 2016). The immediate response provided by the cell of the normal ME is critical to initiating this first line of defense against infection. We found that the cells of the ME have distinctly different potential capacities to contribute immediately to innate immunity. Our results also provide a baseline against which to measure the responses of ME cells to infection in future studies.

### Cell Types of the Normal ME

Seurat PCA analysis of 6,370 ME cells identified 17 cell clusters, each of which displayed a distinct set of genes. The expression of key genes by most of these clusters correspond to cell types that have been observed previously in the ME, while others identify cells not previously known to be present.

#### Epithelial Cells

As noted above, previous authors have proposed five (Lim and Hussl, 1969; Lim, 1979) or six (Hentzer, 1976) morphological categories of ME epithelial cells: basal, intermediate, non-secretory, intermediary secretory, secretory and ciliated epithelial cells. Our transcriptome data are more consistent with five subtypes. Of the five clusters of cells expressing epithelial markers, basal (Cluster 2) and (Cluster 11) ciliated epithelial cell clusters were clearly identified. The three remaining clusters, 0, 1, and 6, were less differentiated by gene expression and presumably less specialized. However, Cluster 1 showed the highest expression of genes encoding secreted factors, including mucins, indicating that this cluster likely consists of fully developed secretory epithelial cells. Secretory genes were expressed at progressively lower levels in Clusters 6 and 0. However, Cluster 0 exclusively expressed surfactant D, also suggestive of an epithelial surface location, and the most innate immune genes of any epithelial type, indicating a prominent role in initial defense of the ME. This cluster may thus represent the morphologically defined “non-secretory” surface epithelial cells. Cluster 6 gene expression was intermediate between that of Clusters 0 and 1, consistent with an intermediate epithelial cell capable of transitioning into either of these two phenotypes.

Regarding the two disparate origins of ME epithelial cells noted by Thompson and Tucker (2013), neural crest versus branchial arch endoderm, there are no recognized adult gene markers for cells of neural crest origin. The small number of melanocytes observed in Cluster 16, which were scattered through epithelial clusters in the Seurat analysis and are assumed to be of neural crest origin (Lin and Zak, 1982), did not express epithelial markers. In addition, we saw no categories of ME epithelial cells that might reasonably be supposed to correspond to this division. It seems likely that dissection of the ME mucosa from the corresponding regions prior to single-cell analysis would be required to determine whether different embryologic origins correspond to any transcriptome and/or functional variations.

#### Stromal Cells

Based on their morphology, ME stromal cells have generally been classified as fibrocytes, with little further distinction. Clusters 3, 4, and 5 were identified as stromal by the expression of many ECM genes. Cluster 5, expressing bone formation genes, appears to correspond to osteoblastic cells. The ME has a strong propensity to generate new bone beneath the stromal layer during OM (Cayé-Thomasen et al., 1999). Cluster 3 was characterized by preferential expression of genes targeting tissue growth, including that of epithelial cells, while cluster 4 expressed genes encoding antimicrobials. These clusters indicate the presence of two additional classes of stromal cells in the ME, cell types not previously recognized on morphological or histochemical grounds.

#### Vascular Cells

Blood vessels, although sparse in the ME, have long been recognized (Lim, 1979). Therefore the vascular endothelial cells of Cluster 8 were to be expected. Morphological studies have also noted the presence of lymphatics in the ME based (Lim and Hussl, 1975), and drainage from the ME to lymph nodes of the neck has been demonstrated (Galich, 1973; Lim and Hussl, 1975), validating lymphatic endothelial cells of Cluster 9 cells. The documentation of capillaries and post-capillary venules in the ME (e.g., Goldie and Hellström, 1990) makes the observation of pericytes in Cluster 13 unremarkable. Gene expression related to hypoxia, contractility and water permeability are consistent with pericyte regulation of vascular tone, based on oxygen tension, and fluid entry into the ME.

#### Leukocytes

Clusters 8 and 9 expressed monocyte markers, while most Cluster 8 cells also expressed macrophage genes consistent with an M2 (alternatively activated) phenotype. Macrophages have been documented in the normal ME (Takahashi et al., 1989; Jecker et al., 1996), but the M2 phenotype has not previously been appreciated. The dendritic cells and less defined monocytes of Cluster 9 have also been noted before in the ME (Jecker et al., 1996). The expression of genes associated with cytotoxicity by dendritic cells indicates a killer phenotype, not recognized previously at this site. Less differentiated monocytes of the classical and resident phenotypes have also not previously been differentiated in the resting ME.

Cells in Clusters 10, 14, and 15 expressed a lymphocyte marker gene *Ptprcap*. Cluster 10 also expressed T-cell and NK-cell genes, while Cluster 15 expressed genes found in B-cells. All three of these lymphocyte types have been reported from the ME (Takahashi et al., 1989). However, the ILC2s of Cluster 14 were not previously reported to be present in the ME.

The number of leukocytes in the normal ME mucosa has often been reported to be relatively low (e.g., Takahashi et al., 1989), and lymphocytes to be especially uncommon (Jecker et al., 1996). However, we observed a substantial number of leukocytes (14.7%) in our normal ME cell samples. This disparity could be caused by differential survival of cells through the tissue digestion and cellular dispersion processes employed. However, it seems unlikely that leukocytes would be dramatically more resistant to these procedures than other cells. Another potential explanation is the diversity of subtypes and the difficulty of definitively identifying leukocytes. For example, many investigators have used the MAC1 antibody, which labels the *Itgam* gene product present in mature macrophages, to identify these cells. However, we found that few macrophages in the normal ME expressed this gene, since the macrophages were likely M2 cells of incomplete maturity.

It should be noted that not all leukocytes known to be present in the ME were found in this study. Several groups (e.g., Watanabe et al., 1991), including our own (Ebmeyer et al., 2005), have documented the presence of mast cells in the normal ME mucosa. Despite this, we were unable to identify any consistent mast cell gene signatures in our cell samples. For example, the entire family of seven mast cell protease (Mcpt) genes showed no expression in any of our ME cells. Other mast cell markers were either absent or were expressed in various cells that had been identified as other cell types with no consistent pattern. The reasons for this are not clear. The number of mast cells in the ME mucosa is not high, so this could represent a sampling issue. Alternatively, the C57BL/6 mouse used in this study may lack ME mast cells, as compared to the guinea pig or WB/B6F1 hybrid mice used in the above studies. However, it seems most likely that mast cells did not survive the cell dissociation method used in this study. The fragility of mast cells isolated from tissues has been noted by others (Kulka and Metcalfe, 2010).

With the exception of mast cells, we were thus able to identify all of the cell types that have been identified previously in the ME using alternative methodologies. In addition, we added new types not previously identified in the normal tympanic cavity. Finally, we obtained a sufficient number of cells for each type to evaluate their individual transcriptomes. This allowed us to achieve our goal of documenting the differences in cell function, as well as the expression of innate immune genes across ME cell types.

### Differential Functions of ME Cell Types

Expression differences identified by clustering analysis and screening of innate immune gene beyond those that specified cell identity, provided clues as to the functional role of cells in the normal ME.

#### Epithelial Cells

The movement of fluids across the mucosal epithelium into and out of the ME is clearly an important aspect of ME homeostasis, OM pathogenesis and fluid clearance during recovery from infection. As indicated by *claudin* gene expression, the cells of the epithelium are joined by tight junctions. Therefore the expression of genes involved in fluid/solute transport across cell membranes by all epithelial clusters is not unexpected, as is expression of the surface liquid layer homeostasis gene *Bipfa1*. Cluster 1 epithelial cells also expressed many genes consistent with presence at the epithelial surface and with secretory activity, including cell-surface and secreted mucins as well as antimicrobials. Cluster 0 similarly expressed antimicrobials, along with surfactant D and ECM-binding genes, suggesting a possible role in basement membrane interactions and that secretory activity is distributed across more than one epithelial cell type. As noted above, Cluster 6 gene expression overlapped with that of Cluster 0 and 1, suggesting an intermediate phenotype. The expression of genes related to tissue proliferation by the basal epithelial cells of Cluster 2 are consistent with them being a source of epithelial cells. However, they also expressed beta defensin 1, further evidence that secretory activity is distributed. Assuming that basal cells are located below the epithelial surface, this antimicrobial may defend against bacteria that invade the mucosa. Cluster 11 ciliated cells expressed *Aqp4* and *Muc4*. Altogether, epithelial cells of the ME mucosa appear specialized for fluid transport and secretory activity.

#### Stromal Cells

The expression of genes by stromal cell clusters was dominated by those related to ECM generation and remodeling. Cluster 3 cells appear also to be specialized for the regulation of epithelial and stromal cell growth, while Cluster 5 cells are likely involved in bone maintenance and remodeling.

#### Vascular Cells

The vascular endothelial cells of Cluster 7 expressed many genes involved in vascular permeability and fluid transport. They therefore work in combination with epithelial cells as the principle regulators of fluid balance in the ME. Not surprisingly, they also expressed genes involved in the recruitment of leukocytes and angiogenesis. Interestingly, vascular and lymphatic endothelial cells each preferentially expressed a different decoy cytokine receptor, which could serve to limit inflammation. Expression of genes involved in hypoxia responses and in regulating vascular tone is typical of pericytes.

#### Leukocytes

Not surprisingly, most of the genes preferentially expressed by leukocytes were involved in immune and inflammatory responses. However, the M2 phenotype and expression of negative immune regulators by the macrophages of Cluster 8 are consistent with a homeostatic and anti-inflammatory role, rather than the pro-inflammatory phenotype of classically activated macrophages. Gene expression by dendritic cells and other monocytes of Cluster 9 was similar, although somewhat fewer genes were preferentially expressed. The lymphocyte types of Clusters 10, 14 and 15 preferentially expressed genes consistent with their expected functions.

### Innate Immune Genes and the Defense of the ME

Infection of the ME activates an innate immune response extremely rapidly (MacArthur et al., 2013; Hernandez et al., 2015). This initial response is likely based largely on the repertoire of innate immune receptor and effector genes that are expressed by the cells of the normal ME. Disruption of many individual innate immune genes in mice has been shown to reduce the effectiveness of this innate response to infection, and to prolong OM. Moreover, several studies have identified polymorphisms in human innate immune genes that are linked to otitis media proneness in patients (see Kurabi et al., 2016 for a recent review). These studies underscore the importance of innate immunity in the immediate and long-term defense of the ME, which was our rationale for focusing on these genes in ME cell transcriptomes.

As can be seen from Figure 5, expression of innate immune genes is distributed across the various cell types of the ME. Epithelial cells appear specialized for the detection and response to pathogens, including the secretion of antibacterial peptides. However, they also expressed genes related to leukocyte recruitment and activation.

Stromal cells expressed several complement factors and pro-inflammatory genes, suggesting potential involvement in active inflammation. They also expressed many negative immune regulators, not only of complement but also of innate immunity in general as well as T- and NK-cells. They thus appear to serve as inhibitory modulators of inflammation to a greater extent than epithelial cells.

Endothelial cells and pericytes expressed several genes involved in the activation of inflammatory pathways and T-cells, but also several negative immune regulators.

The M2 macrophages in the ME expressed by far the most innate immune genes, with an emphasis on pathogen detection, leukocyte chemotaxis, complement activation, and both positive and negative inflammatory regulation. Their phenotype is consistent with immune homeostasis, but also readiness to act in case of infection. Monocytes/dendritic cell gene expression was similar to that seen in macrophages, but cytotoxic genes were additionally expressed.

The lymphocytes of cluster 10 were primarily gamma-delta T-cells that typically have an innate immune function, and consequently expressed several pathogen receptors and innate immune effectors. Cytotoxic T-cells and NK-cells were also present in this cluster. The ILC2s of cluster 14 expressed several genes involved in T-cell regulation and induction of inflammation. The B-cells of cluster 15, as expected, expressed genes related to antibody production.

In addition to individual genes, the GO: biological process gene categories that were most regulated in macrophages and monocytes are all subcategories of the GO category for innate immunity. For the remaining ME cell types, innate immunity was not among the GO categories with the highest degree of regulation. This is not surprising, since unlike macrophages and monocytes, the primary functions of these cells are not innate immunity. However, the fact that they also express many innate immune genes underscores the importance of innate immunity in virtually all ME cell types.

A striking feature of innate immune gene expression in normal ME cells was the large number of negative regulators of immunity and inflammation observed in the transcriptomes of many different cell types, in parallel with to pro-inflammatory genes. This pattern of expression suggests that, while the cells of the ME are primed to respond to infection by generating inflammation and other antimicrobial responses, these processes are actively held in check by immune inhibitory genes to ensure ME mucosal homeostasis. This not only must pro-inflammatory gene products be activated by pathogens at the initiation of OM, anti-inflammatory genes may need to be downregulated to allow maximally effective innate immunity to defend the ME. Other investigators have noted the importance of endogenous negative regulators in ameliorating OM Pathogenesis (e.g., Li et al., CYLD). Our results add a large number of additional negative regulators which were distributed across diverse ME cell types.

### Limitations of the Study

Our analysis of single-cell transcriptomes is subject to a number of methodological limitations. As noted above, the numbers of different cell types recovered is dependent upon the survival of cells through the enzymatic digestion and dispersion techniques. While we were able to document an effect of this for mast cells, the extent to which selection bias may have influenced our other cell populations is not clear. Fortunately, we obtained sufficient numbers of cells in all cell populations to adequately survey gene transcription. However, the proportions of each cell type in our samples should not be taken to represent their relative abundance *in vivo*.

Another limiting effect is related to the limited number of genes that can be recovered from an individual cell. This means that abundant mRNAs are more likely to be represented than scarce transcripts. It has been argued that the detection of scarce mRNAs is stochastic, and that combining the results of 20 or more cells approaches the results of bulk RNASeq (Marinov et al., 2014). However, the possibility that scarcer transcripts were missed in a given cell population must be considered.

Other limitations reflect the nature of gene responses to infection. It is of course certain that the expression of innate immune and other genes by ME cells would change dramatically upon infection, and that some of these changes would be very rapid (MacArthur et al., 2013; Hernandez et al., 2015). Therefore, the roles of ME cells in the initial stage of OM, and beyond, would correspondingly change. Our data reflect only pre-existing mRNAs that may be expressed in readiness for a pathogen challenge. We plan to assess the results of infection on the transcriptomes of ME cell types. This work is in progress, and will be the basis of a future report. We felt that including results from infected ME cells would have made this paper unacceptably long.

## Conclusion

The results of this study establish, for the first time, the differences that exist between the transcriptomes of cells in the normal ME. They identify not only previously known cell types, but also show the presence of novel cell types and subtypes. The genes that differentiate these cell types provide information on their roles in ME homeostasis, and their ability to respond immediately to infection. The results also provide a baseline from which to assess the molecular responses of ME cells to infection.

## Data Availability Statement

The single cell transcriptome data is available in GEO (GSE146244, https://www.ncbi.nlm.nih.gov/geo/query/acc.cgi?acc=GSE146244).

## Ethics Statement

The animal study was reviewed and approved by the Institutional Animal Care and Use Committee of the VA San Diego Medical Center.

## Author Contributions

AR and AK wrote the main manuscript. KP, CD, and AK preformed the wet laboratory experiments. CN, KF, and NW performed the bioinformatics analyses. AR and CN generated the figures and table. All authors reviewed the final version of the manuscript.

## Conflict of Interest

AR is a co-founder of Otonomy Inc., serves as a member of the Scientific Advisory Board, and holds an equity position in the company. The UCSD Committee on Conflict of Interest has approved this relationship. Otonomy, Inc., played no part in the research reported here. The remaining authors declare that the research was conducted in the absence of any commercial or financial relationships that could be construed as a potential conflict of interest.

## Supplementary Material

FIGURE S1 A heat map illustrating the expression of the 520 differentially expressed genes, arranged alphabetically, across ME cell clusters.
