# Supplementary figures and images for: Single-Cell Transcriptomes Reveal a Complex Cellular Landscape in the Middle Ear and Differential Capacities for Acute Response to Infection
Source: Front Genet. 2020 Apr 15;11:358. doi: 10.3389/fgene.2020.00358 (PMC7174727; doi:10.3389/fgene.2020.00358)

Supplementary Figure 1

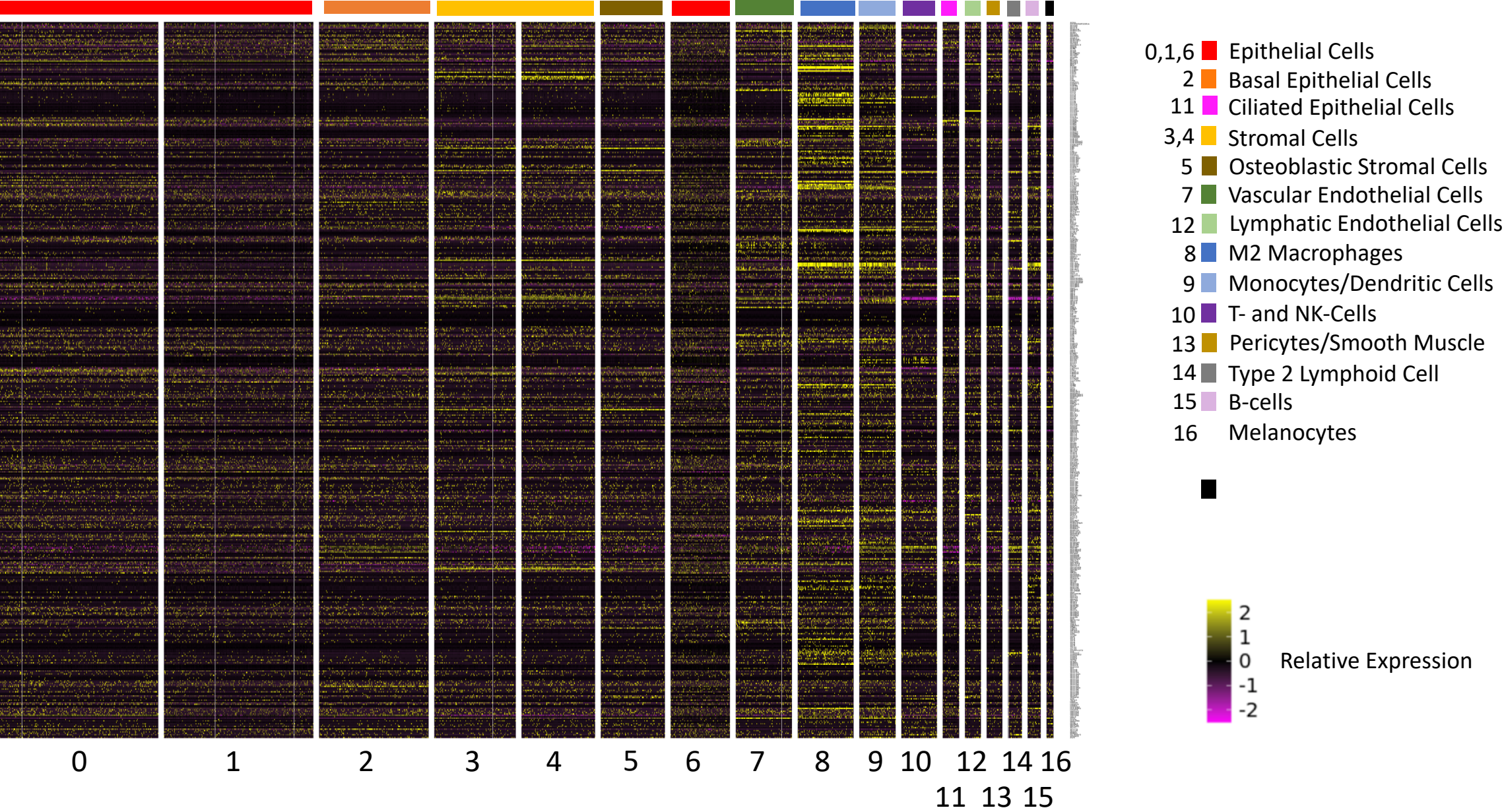

Supplement: FIGURE S1 — A heat map illustrating the expression of the 520 differentially expressed genes, arranged alphabetically, across ME cell clusters. [file Data_Sheet_1.PDF]
